# Circulating angiogenic factors in diabetes patients in a tertiary hospital in Ghana

**DOI:** 10.1186/s40200-016-0267-1

**Published:** 2016-10-10

**Authors:** Kwame Yeboah, Eric Kyei-Baafour, Daniel A. Antwi, Henry Asare-Anane, Ben Gyan, Albert G. B. Amoah

**Affiliations:** 1 Department of Physiology, School of Biomedical and Allied Health Sciences, University of Ghana, P O Box KB143, Korle-Bu, Accra, Ghana; 2 Department of Chemical Pathology, School of Biomedical and Allied Health Sciences, University of Ghana, Accra, Ghana; 3 Department of Medicine & Therapeutics, School of Medicine and Dentistry, University of Ghana, Accra, Ghana; 4 Department of Immunology, Noguchi Memorial Institute of Medical Research, University of Ghana, Accra, Ghana; 5 National Diabetes Management & Research Centre, Korle-Bu Teaching Hospital, Accra, Ghana

**Keywords:** Angiopoietins, Estimated glomerular filtration rate, Diabetes, Hypertension, Ghana

## Abstract

**Background:**

Impaired angiogenesis is amongst the underlining mechanisms of organ damage in diabetes and hypertensive patients. In diabetes and hypertensive patients without proteinuria and overt CVDs, we studied the levels of angiogenic growth factors, angiopoietin (Ang)-1, Ang-2 and vascular endothelial growth factor (VEGF), and the relationship between these angiogenic growth factors and renal function, measured as estimated glomerular filtration rate (eGFR).

**Method:**

In a case control design, 107 type 2 diabetes (T2DM) patients and 93 non-diabetes controls were recruited into the study. Levels of plasma glucose, lipids, creatinine and angiogenic growth factors; Ang-1, Ang-2 and VEGF measured from fasting blood samples. Estimated glomerular filtration rate (eGFR) was computed using Chronic Kidney Disease Epidemiology Collaboration (CKD-EPI) algorithm and eGFR < 60 ml/min/1.73 m^2^ was considered to be low. Multivariable logistic regression was used to assess the odds of change in angiogenic growth factors among patients with diabetes and hypertension, and patients with low eGFR, compared to those without these conditions.

**Results:**

In a total of 200 participants with 49 % females and mean age of 54.1 ± 10.2 years, 22.7 % of T2DM patients and 13.3 % of non-diabetes participant had low eGFR. The levels of Ang-1 and Ang-2 were highest in hypertensive T2DM patients, followed by patients with either T2DM or hypertension alone, with the controls having the lowest levels. The odds of change in circulating Ang-2 levels increased in patients with both diabetes and hypertension [11.76 (7.97–16.63), *p* < 0.01] compared to patients with either diabetes [5.45 (3.31–9.71), *p* = 0.02] or hypertension [5.45 (3.31–9.71), *p* = 0.02] alone. Compared to those with normal eGFR, the odds of change in serum Ang-2 levels were increased in patients with low eGFR in both the crude [1.26 (1.08–2.110), *p* = 0.023] and adjusted [1.14 (1.03–2.34), *p* = 0.043] regression models.

**Conclusion:**

In our study population, having diabetes and hypertension increased the levels of Ang-1 and Ang-2. Also, low eGFR status was associated with increased levels of Ang-2 after adjustment for other risk factors.

## Background

Type 2 diabetes (T2DM) and hypertension are expected to soon reach epidemic levels in sub-Saharan African populations. Both diabetes and hypertension are associated with early and aggressive forms of macro- and micro-vascular diseases in all population [1–4]. Impaired angiogenesis has been reported to be one of the major mechanistic pathways that culminates in premature cardiovascular diseases (CVDs) in diabetes and hypertensive patients [5, 6]. Angiogenesis is known to be regulated by circulating angiogenic growth factors, which have trophic and proliferative effects on the microvessels [7]. The best-studied angiogenic factors are vascular endothelial growth factor (VEGF), angiopoietin 1 (Ang-1) and angiopoietin 2 (Ang-2) [8]. The primary source of Ang-1 is non-endothelial cells, including pericytes (peri-endothelial cells), whereas Ang-2 is predominantly expressed in endothelial cells, stored in vesicles known as Weibel-Palade bodies, and is rapidly released in response to specific stimuli [7]. Both Ang-1 and Ang-2 are ligands for Tie2 receptor which is expressed on the endothelial cells. Emerging evidence indicates that hyperglycaemia modulates the levels of circulating angiogenic growth factors, which may explain their important role in the pathogenesis of organ damage in diabetes patients [5, 6, 9]. Angiopoietins promote angiogenesis together with vascular endothelial growth factor (VEGF) [9–11].

In this study, we investigated the levels of circulating angiogenic growth factors in diabetes and hypertensive patients without overt CVDs or proteinuria, and the relationship between angiogenic growth factors and renal function. We hypothesized that, compared to the individual diseases, coexistence of diabetes and hypertension will elevate the levels of angiogenic factors. Also, low eGFR status, indicative of renal dysfunction, will be associated with imbalance in the levels of angiogenic growth factors.

## Methods

### Study design and subjects

The study was conducted at the National Diabetes Management and Research Centre, Accra, Ghana; which is an ambulatory clinic for diabetes research, management and education. In all, 200 participants comprising 107 T2DM patients and age- and gender- matched 93 non-diabetes volunteers, aged 35–75 years, were recruited into the study. T2DM status was diagnosed clinically, based on the patient being diagnosed at older age (>35 years) and managed with non-insulin therapy for at least 2 years. Based on gender and age-decade of T2DM patients, non-diabetes volunteers from the surrounding communities were randomly recruited and screened for diabetes using oral glucose tolerance test; fasting plasma glucose ≤7 mmol/l and 2-h post-glucose load plasma glucose ≤11.2 mmol/l. Due to the effect of high blood pressure (BP) on angiogenic growth factors [12], the study participants were categorised based on their hypertension status, defined as BP > 140/90 mmHg and/or use of antihypertensive medication. Individuals with foot ulcers, established CVDs and proteinuria (+1 or more in urine dipstick) were excluded from the study. Ethical approval for this study was given by the University of Ghana Medical School Ethical and Protocol Review Committee (Protocol ID number: MS-Et/M.2 – P.4.10/2012–2013) and all participants provided written informed consent after the procedures involved in the study were thoroughly explained to them.

### Anthropometric measurements

Using standard procedures [13], waist and hip circumferences were measured in duplicate with a non-elastic tape, maximum height to the nearest 0.1 cm using a clinical Stadiometer, and weight to the nearest 0.1 kg on a digital, heavy-duty floor scale (both from Secca, Hamburg, Germany). Body composition monitor (BF- 508, Omron Healthcare, Inc., Vernon Hills, IL, USA) was used to assess percentage body and visceral fat.

### Biochemical analysis

Blood samples were drawn in the morning, after 8–12 h of overnight fasting. Fasting plasma glucose (FPG), 2-h post glucose load plasma glucose (2-h PPG), total cholesterol (TC), high-density lipoprotein cholesterol (HDL), triglyceride (TG) and serum creatinine levels were analysed using BS 400 chemical autoanalyser (Mindray, China) and commercial reagents (Randox Laboratory Reagents, UK). Low-density lipoprotein cholesterol levels were calculated using Friedewald’s formula. eGFR was calculated using CKD-EPI algorithm and eGFR <60 ml/min/1.73 m^2^ was considered to be low.

### Angiopoietins and VEGF assays

Serum levels of Ang-1, Ang-2 and VEGF by sandwich enzyme-linked immunosorbent assay using commercially available enzyme-linked immunosorbent assay kits (R&D Systems, Minneapolis, MN). The assays were performed according to the manufacturer’s recommendations and the total inter-assay coefficient of variation for the three assays were <7 %. Lowest limit of the detection were 0.03 ng/ml for VEGF, 0.16 ng/ml for Ang-1 and 0.06 ng/ml for Ang-2.

### Statistical analysis

Continuous data were analysed with the Shapiro-Wilk test to determine their distribution. Variables with normal distribution were presented as mean ± standard deviation and analysed using ANOVA. Variables with non-normal distribution were presented as median & interquartile range and analysed using Kruskall-Wallis test. Categorical data were analysed by the *χ* ^2^ test. Spearman’s correlation coefficients were computed to assess the association between the angiogenic factors and clinical and metabolic parameters. Multivariable logistic regression models were performed to compute adjusted odd ratios between (1) angiogenic growth factors versus diabetes and/or hypertension, and (2) angiogenic growth factors versus eGFR status.

## Results

There were no differences in the mean values of age, gender distribution, alcohol intake, previous smoking status, BMI, body fat, visceral fat, HDL cholesterol and serum triglycerides. T2DM subjects, irrespective of hypertension status, had higher levels of total cholesterol and LDL cholesterol than non-diabetes participants. As expected, BPs of hypertensive patients were higher than non-hypertensive patients, irrespective of diabetes status. Creatinine levels were highest in hypertensive T2DM patients and patients with hypertension only, followed by patients with T2DM only, with the controls having the lowest level. eGFR was lowest in hypertensive T2DM patients, followed by hypertension only and T2DM only patients with similar eGFR levels, and controls had the highest eGFR levels. The overall prevalence of low eGFR was 18.1 % (Table 1). Ang-1 levels were elevated hypertensive T2DM patients. Compared with control subjects, Ang-2 levels were highest in hypertensive T2DM patients, followed by patients with diabetes only or hypertension only (Table 2). When the participants were dichotomised based on diabetes status, the serum levels of Ang-1 [42.7 (27–57.1) vs. 37.2 (28.1–43) nmol/ml, *p* = 0.027], Ang-2 [0.85 (0.48–1.25) vs. 0.6 (0.27–1) nmol/ml, *p* = 0.011] and VEGF [75.7 (30.7–208.1) vs. 50.2 (17.4–110.1) pmol/ml, *p* = 0.022] were higher in T2DM patients than non-diabetes participants respectively. However, on the basis of hypertension status, only Ang-2 [0.8 (0.42–1.3) vs. 0.58 (0.4–1.02) nmol/ml, *p* = 0.022] was higher in hypertensive patients than non-hypertensive participants respectively.

**Table 1:** Characteristics of study participants

Characteristics	Hypertensive T2DM (*n* = 63)	T2DM only (*n* = 44)	Hypertension only (*n* = 54)	Controls (*n* = 39)
Age	54.5 ± 10.8	52.6 ± 8.9	54.6 ± 9.4	50.9 ± 11.1
Females, n (%)	33 (52.3)	15 (34.1)	31 (57.4)	19 (48.7)
Alcohol intake, n (%)	21 (31.7)	28 (63.6)	13 (24.1)	21 (53.4)
Previous smokers, n (%)	6 (9.5)	8 (18.2)	11 (20.4)	12 (30.8)
Duration of diabetes, yrs	9.95 ± 7.36^c^	6.59 ± 5.86		
BMI, kg/m^2^	30.7 ± 6.2	26.3 ± 4.3	29.8 ± 5.5	28.7 ± 5.3
Body fat, %	36.2 ± 12.9	30.3 ± 10.2	37.8 ± 11.8	32.2 ± 13.2
Visceral fat, %	12.4 ± 4.7	10.8 ± 4.8	10.7 ± 3.2	10.2 ± 3.7
Waist circumference, cm	103 ± 13	94 ± 10	97 ± 11	92 ± 21
Waist-hip ratio	0.92 ± 0.07	0.91 ± 0.07	0.90 ± 0.07	0.90 ± 0.21
Systolic BP, mm Hg	153 ± 28^ac^	124 ± 9	142 ± 40^ac^	123 ± 10
Diastolic BP, mm Hg	90 ± 13^ac^	74 ± 7	88 ± 13^ac^	73 ± 9
Pulse BP, mm Hg	65 ± 13^ac^	50 ± 7	63 ± 14^ac^	50 ± 7
Mean BP, mm Hg	111 ± 14^ac^	91 ± 7	109 ± 14^ac^	90 ± 8
Heart rate, per min	75 ± 15	74 ± 10	65 ± 23	66 ± 11
FPG, mmol/L	7.8 ± 2.3^ab^	9.1 ± 3.5^ab^	4.9 ± 2.9	5 ± 1.3
2 h-PPG, mmol/L			8.2 ± 4.8	5.2 ± 2.1
Total cholesterol mmol/L	4.4 ± 1.4^ab^	4.1 ± 1.3^ab^	5.2 ± 1.3	5.3 ± 1.2
Triglycerides, mmol/L	1.3 ± 0.5	1.1 ± 0.5	1.2 ± 0.6	1.1 ± 0.4
HDL, mmol/L	0.7 ± 0.2	0.8 ± 0.1	1 ± 0.3	1.2 ± 0.2
LDL, mmol/L	3.9 ± 1.3^ab^	3.82 ± 1.27^ab^	3.20 ± 1.63	3.1 ± 1.38
Serum creatinine, mg/dL	1.42 (1.05–3.76)^ac^	1.23 (1.01–2.06)^a^	1.18 (1.02–2.27)^a^	1.03 (0.98–1.67)
eGFR ml/min/1.78 m^2^	71.7 (54.2–81.4) ^abc^	88.1 (68.3–84.7)^a^	81.2 (57.9–91.2)^a^	108.6 (87.9–121.6)
eGFR < 60, n (%)	19 (30.2)	6 (13.6)	11 (20.4)	2 (5.1)

*T2DM* type 2 diabetes, *BMI* body mass index, *BP* blood pressure, *FPG* fasting plasma glucose, *2 h-PPG* 2-h post glucose-load plasma glucose, *TC* cholesterol, *TG* triglycerides, *LDL* low density lipoprotein cholesterol, *HDL* high-density lipoprotein cholesterol, *eGFR* estimated glomerular filtration rate

^a^ vs controls, *p* < 0.05

^b^ vs hypertensive only, *p* < 0.05

^c^ vs T2DM only, *p* < 0.05

**Table 2:** Plasma levels of vascular growth factors

	Hypertensive T2DM (*n* = 63)	T2DM only (*n* = 44)	Hypertension only (*n* = 54)	Controls (*n* = 39)	p
Ang-1 (ng/mL)	44.3 (30.4–62.2)^a^	37.3 (24.9–46.5)	36.1 (23.7–43)	36.3 (27.3–41)	0.02
Ang-2 (pg/mL)	875.6 (515.2–1482.4)^ab^	710.4 (419–1084.1)^a^	764.4 (390.8–1128)^a^	467.6 (180–877.7)	<0.01
VEGF (pg/mL)	108.1 (42.7–206.5)	54.4 (15.4–197.1)	51.7 (11.9–118.4)	47.2 (22.1–104.5)	0.05

*T2DM* type 2 diabetes, *NDM* non-diabetes, *ang-1* angiopoietin 1, *ang-2* angiopoietin 2, *VEGF* vascular endothelial growth factor

^a^ vs controls, *p* < 0.05

^b^ vs hypertension only, *p* < 0.05

In univariate correlational analysis, Ang-1 levels correlated positively with BMI, body fat, waist circumference, FPG, systolic and pulse BPs. Ang-2 levels also correlated positively with BMI, body fat, waist circumference, systolic, diastolic, pulse & mean BPs, but negatively associated with duration of diabetes. There were no correlation between VEGF and any of the clinical variables (Table 3).

**Table 3:** Association between vascular growth factors and patient’s characteristics

	Ang-1		Ang-2		VEGF	
	r	p	r	p	r	P
Age	−0.03	0.65	0.05	0.54	0.08	0.26
Duration of diabetes	−0.03	0.79	−0.27	0.01	−0.03	0.79
BMI	0.18	0.01	0.21	<0.01	0.01	0.91
Body fat	0.14	0.05	0.24	<0.01	0.03	0.72
Visceral fat	0.05	0.46	0.06	0.42	0.03	0.71
Waist circumference	0.16	0.03	0.20	<0.01	0.03	0.68
Waist-hip ratio	0.03	0.69	0.01	0.87	0.05	0.54
FPG	0.21	<0.01	0.15	0.04	0.09	0.36
2 h-PPG	0.06	0.52	−0.03	0.86	−0.01	0.97
TG	0.10	0.62	0.06	0.44	−0.05	0.49
TC	0.04	0.62	−0.09	0.25	0.02	0.80
HDL	−0.04	0.60	−0.02	0.75	0.01	0.90
LDL	0.05	0.49	−0.10	0.19	0.02	0.81
Systolic BP	0.15	0.04	0.25	<0.01	0.11	0.14
Diastolic BP	0.07	0.33	0.19	0.01	0.02	0.75
Pulse BP	0.18	0.01	0.24	<0.01	0.11	0.14
Mean BP	0.12	0.11	0.23	<0.01	0.05	0.52

*T2DM* type 2 diabetes, *NDM* non-diabetes, *BMI* body mass index, *SBP* systolic blood pressure, *DBP* diastolic blood pressure, *PP* pulse pressure, *MBP* mean blood pressure, *HR* heart rate, *FPG* fasting plasma glucose, *2 h-PPG* 2-h post glucose-load plasma glucose, *TC* cholesterol, *TG* triglycerides, *LDL* low density lipoprotein cholesterol, *HDL* high-density lipoprotein cholesterol

**Fig. 1:**
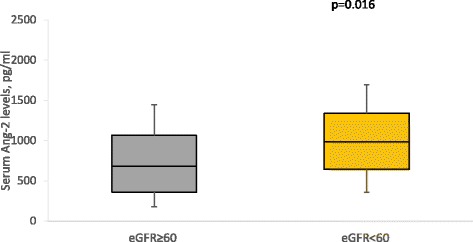
Serum Ang-2 Levels by eGFR status

**Fig. 2:**
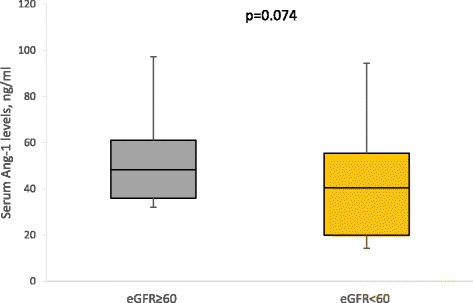
Serum Ang-1 Levels by eGFR status

**Fig. 3:**
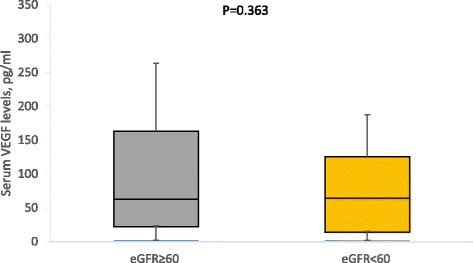
Serum VEGF Levels by eGFR status

In multivariable logistic regression models, compared to the healthy controls, the odds of change in Ang-2 significantly increased 4.6 times, 5.5 times and 11.8 times in patients with only hypertensive, patients with only T2DM and hypertensive T2DM patients, respectively. The odds of change in Ang-1 levels increased in hypertensive T2DM patients (Table 4). When the study participants were categorised based on renal function, patients with low eGFR (<60 ml/min/1.78 m^2^) had higher levels of Ang-2 than those with normal eGFR (≥60 ml/min/1.73 m^2^) (Fig. 1). There were no difference in the levels of Ang-1 (Fig. 2) and VEGF (Fig. 3) by eGFR status. Compared to participants with normal eGFR, low eGFR status was associated with increase odds of change in Ang-2 levels, both in unadjusted and multivariable logistic regression models (Table 5).

**Table 4:** Multivariable logistics regression diabetes/hypertension status and angiogenic factors

	logAng-1		logAng-2		logVEGF	
	OR (95 % CI)	p	OR (95 % CI)	p	OR (95 % CI)	p
Hypertension only	1.21 (0.79–1.41	0.58	4.57 (2.15–8.13)	0.03	1.02 (0.54–1.96)	0.94
T2DM only	1.1 (0.89–1.21)	0.60	5.45 (3.31–9.71)	0.02	1.26 (0.64–2.49)	0.50
Hypertensive T2DM	1.11 (1.06–1.31)	0.04	11.76 (7.97–16.63)	<0.01	1.89 (0.98–3.87)	0.09

Adjusted for age, gender, diabetes & hypertension status, diabetes and antihypertensive medication, mean BP, BMI and WHR

*OR* odds ratio, *T2DM* type 2 diabetes, *NDM* non-diabetes, *ang-1* angiopoietin 1, *ang-2* angiopoietin 2, *VEGF* vascular endothelial growth factor

**Table 5:** Multivariable logistic regression of angiogenic growth factors and low eGFR

	Unadjusted model		Adjusted model^a^	
	OR (95 % CI)	p	OR (95 % CI)	p
logAng-1	0.97 (0.54–3.95)	0.379	1.16 (0.72–4.91)	0.488
logAng-2	1.26 (1.08–2.110)	0.023	1.14 (1.03–2.34)	0.043
logVEGF	1.17 (0.65–2.12)	0.199	1.12 (0.66–2.24)	0.232

^a^Adjusted for age, gender, diabetes & hypertension status, diabetes and antihypertensive medication, mean BP, BMI and WHR

## Discussion

The major findings of this study are two-folds; (1) angiogenic factors, particularly Ang-2, is elevated in diabetes and hypertension patients and (2) Ang-2 is increased in patients with eGFR < 60 ml/min/1.73 m^2^. The ASCOT study [12], involving older Caucasians, reported elevation of Ang-1, Ang-2 and VEGF in hypertensive patients compared to non-hypertensive subjects. However, the inclusion of 36 % and 43 % of diabetes patients that in both normotensive and hypertensive groups respectively, as was acknowledged by the authors, confounded the interpretation of the results. In our study therefore, we stratified the study population based on diabetes and hypertension status.

Other studies reported increased levels of Ang-1 and Ang-2 in diabetes patients. For instance, in older Caucasian diabetes population in the United Kingdom, Ang-2 and VEGF levels were elevated irrespective of the presence or absence of CVDs [14]. In Austrian diabetes patients, the presence of micro- and macro-vascular complications was associated with increased Ang-2 levels [11]. Contrary to our findings, the levels of Ang-1 decreased in South Asian middle-aged diabetes patients and Ang-1 and Ang-2 were both decreased Caucasian diabetes patients with/without CVDs [15]. The small sample size (<25 patients per arm) of this study might be the major explanation for not detecting any significant difference in the angiogenic growth factor. Population-based studies might be needed to resolve these inconsistencies in the levels of angiogenic growth factors in T2DM patients, especially in sub-Saharan African population.

In this study, univariate and multivariate logistic analysis showed that low eGFR, indicative of renal impairment, is associated with elevation in Ang-2 levels. In agreement with our findings, Ang-2 increased with severity of renal dysfunction in untreated, non-smokers with CKD. Also, in healthy individuals, Ang-2 increased after unilateral nephrectomy [16]. In CKD patients on dialysis with elevated Ang-2 levels, kidney transplant reversed Ang-2 levels to normalcy. In addition, longitudinal study has shown than Ang-2 can predict mortality in CKD patients [17]. In a population study as well, Ang-2 predicted all-cause mortality and CVD-specific mortality [18].

The findings of the present study also indicate that important CVD risk factors such as BMI, body fat, waist circumference, systolic and pulse BPs were correlated with Ang-1 and Ang-2 but not with VEGF. Other studies have also shown that adiposity has influence on angiogenic factors and ultimately angiogenesis [19, 20]. Also, in multivariate logistic regression analysis, Ang-2 was the only angiogenic factor that was significantly associated with diabetes and hypertension status. Ang-2 has been reported to be independently associated with CVDs in longitudinal and cross-sectional studies [10]. In Chinese diabetes patients, Ang-2 was independently associated with the degree of left ventricular dysfunction in patients with unstable angina pectoris [21]. Iribarren et al. (2011) also reported association of Ang-2 with erectile dysfunction. In a nested case–control study of patients with acute myocardial infarction, a unit increase in serum Ang-2 level was associated with 1.17 increased odds of myocardial infarction [5, 6].

The physiological mechanisms underlying the activities of Ang-1, Ang-2 and VEGF indicate that Ang-1 promotes circumferential growth required for vessel maturation and stabilization, whereas Ang-2/VEGF promotes vessel proliferation. In mouse embryos lacking Ang-1 or Tie2, the initial growth of the vasculature is interrupted by failure to undergo the normal remodelling process. The dysfunction of pancreatic β cells, as occurred in diabetes and insulin resistance state, is associated with impaired levels of angiogenic factors. In insulin resistance state, there is enlargement and reduction of capillaries of β cells without alteration in the expression of angiogenic factors [22]. However, in diabetes, hyperglycaemia triggers over-expression of Ang-2, leading to the disorganisation and reduction in pancreatic capillary density [23].

The limitations for this study include cross sectional data collected from hospital-based diabetes patients from a tertiary health centre; this limit the generalizability of the results to the entire diabetes population in Ghana. Although the participants in this study had no history or overt CVD, we cannot rule out the presence of sub-clinical CVDs, and hence, we may interpret the results cautiously. Future studies might utilize a longitudinal population-based design with adequate screening of subclinical CVDs to investigate the role of angiogenic factors in diabetes and hypertension patients.

## Conclusion

In summary, the current study provides evidence that in T2DM patients of black ethnicity in our study population, there is impairment in factors regulating angiogenesis. Also, Ang-2 renal dysfunction was associated with impairment in agiogenesis. This suggest that Ang-2 might be a potential biomarker linking diabetes and hypertension to renal dysfunction. However, future longitudinal studies have to clarify the role of Ang-2 in renal homeostasis in diabetes and hypertension patients in sub-Saharan Africa. This might ultimately pave the way for therapeutic interventions to alleviate the burden of diabetes-associated renal disease in blacks.

## Abbreviations

Ang: Angiopoietin
CVDs: Cardiovascular diseases
eGFR: Estimated glomerular filtration rate
T2DM: Type 2 diabetes
VEGF: Vascular endothelial growth factor
